# Study on the treatment of sudden cadmium pollution in surface water by a polymer enhanced ultrafiltration process†

**DOI:** 10.1039/d0ra10818a

**Published:** 2021-02-12

**Authors:** Qian Meng, Jun Nan, Yuxi Mu, Xuehui Zu, Mingqi Guo

**Affiliations:** State Key Laboratory of Urban Water Resource and Environment, School of Environment, Harbin Institute of Technology No. 73, Huanghe Road Harbin 150090 PR China nanjun219@163.com +86 451 86283001 +86 451 86084169

## Abstract

The removal of cadmium(ii) pollution in surface water by a polymer enhanced ultrafiltration (PEUF) process was investigated. Three water soluble polymers, chitosan (CTS), polyvinyl alcohol (PVA) and polyacrylate sodium (PAAS), were selected for this study. Of the three polymers, PAAS had strong interactions with Cd^2+^, and the PEUF achieved a high removal of Cd^2+^; therefore, PAAS was used as a complexing agent for the simulated cadmium pollution experiment. Experiments were performed as a function of the aqueous pH, polymer/Cd^2+^ ratio (P/M), ionic strength and humic acid. Under optimum experimental conditions, the Cd^2+^ removal rate reached 100%. pH was the main factor affecting removal of Cd^2+^, which decreased to 60% at a pH of 4. The Cd^2+^ removal was found to decrease as NaCl and HA were added. The analysis showed that the mechanism of NaCl could be a compressed electric double layer, while the mechanism of HA and H^+^ was competitive complexation. Finally, UF membrane fouling, the dissociation of PAAS–Cd and the regeneration of PAAS were investigated. The results showed that the higher P/M was, the lower the pH, the higher the HA concentration, and the more serious the UF membrane fouling were. The dissociation rate of PAAS–Cd reached 99.8% at a pH of 2.5. When the P/M was 5, the removal rate of Cd reached 99.6% with the addition of 20% new PAAS in the regenerate. This result showed that the PEUF process could be a promising method for removing Cd pollution in surface water.

## Abstract 2

The removal of cadmium(ii) pollution in surface water by a polymer enhanced ultrafiltration (PEUF) process was investigated.

## Graphical abstract

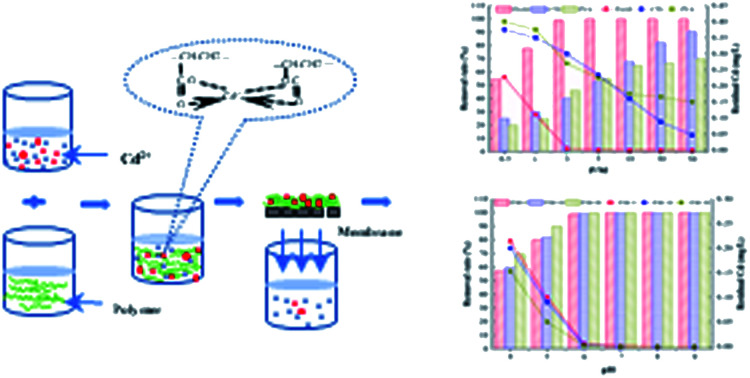

## 1 Introduction

Cadmium (Cd) is a common heavy metal in polluted water that mainly comes from the illegal discharging of cadmium deposits, nonferrous metal metallurgy and the electroplating process.^1–3^ Contamination accidents caused by Cd are the most severe and common pollution incident. Therefore, Cd is also referred to as the most toxic element among all heavy metals. In addition, Cd is extremely noxious to human health.^4–6^ The European Union, America and China all have strict standards for heavy metal in drinking water, among which the concentration of cadmium should be lower than 0.005 mg L^−1^ in China drinking water standard.^7^ In general, the addition of alkali to polluted water is the dominant method to treat Cd pollution. However, a main drawback restricting the application of this technology in drinking water treatment is the long treatment time. Hence, it is urgent to explore an efficient Cd removal method with a short treatment time that properly matches traditional water treatment techniques.

Polymer enhanced ultrafiltration (PEUF), a new technology developed in recent years, can concentrate and purify heavy-metal-polluted water and has been widely applied in industrial wastewater treatment. This technology has attracted much interest due to its many advantages, such as its excellent effectiveness, easy accessibility and saving of space.^8–10^ The PEUF process is based on the principle that water-soluble polymers with molecular weights larger than the molecular weight cut-off (MWCO) of UF membranes can bind heavy metals to form macromolecular complexes and be retained by UF membranes. The concentrated retentate composed of the water-soluble polymer and the small sized heavy metals may be subjected to further treatment for recovery of the heavy metals and reuse of the polymer if needed. After ultrafiltration, there are almost no free metal ions in the permeation. Many studies have revealed a high removal rate of metal ions by PEUF.^11,12^ For instance, Yifeng Huang found that using polyethyleneimine (PEI), polyvinyl alcohol (PVA) and polyacrylic acid (PAA) as polymers to remove mercury(ii) ions led to removal rates that exceeded 90%.^13^ Jiahui Shao used sodium polyacrylate (PAAS) and PEI as polymers and obtained nickel removal rates of 99.5% and 93.0%,^14^ respectively. Researchers have achieved a 100% removal rate of chromium.^15^ However, most previous research has focused on the application of PEUF technology to treat high-concentration Cd in heavy metal industrial wastewater. Little attention has been paid to the application of PEUF techniques for the removal of Cd pollution in surface water. Therefore, the feasibility of using PEUF to deal with simulated Cd pollution in surface water was investigated in this study.

Complexation is the main chemical reaction in the PEUF process to remove metal ions. This process is affected by the kinds of agents, the hydraulic conditions and the properties of the aqueous solution. A suitable polymer is the key to the use of PEUF technology in the drinking water treatment process. Therefore, the following points need to be taken into account in PEUF: (I) the permeate meets the safe drinking standard; (II) the water-soluble polymer is nontoxic and cannot leak; (III) the polymer is economical, accessible and recyclable. Frequently used macromolecular organic polymers include PEI, PVA, chitosan (CTS), carboxymethylcellulose, PAAS and sodium alga acid. In addition to the type of polymer, the mass concentration ratio between the polymer and metal ions (P/M), the solution chemical, the competitive complexing agent, and the feed water flow rate and transmembrane pressure difference could affect the removal of metal ions in PEUF.^16–19^ However, the solution pH and the ionic strength are the most important factors to be considered in the treatment of drinking water. The solution pH affects not only the physicochemical properties of the polymer but also the interaction between the polymer and the metal ions, as well as the surface charge of the UF membrane. When the solution pH increases, the metal ions removal rate of PEUF increases.^12,20^ The ionic strength also has a great influence on the removal rate.^21,22^ Na^+^ affects not only the physicochemical properties of the polymer but also the effectiveness of PEUF in removing heavy metal ions. In addition, natural organic matters such as humic acid (HA) in surface water can influence the interaction between the polymers and the metal ions. HA is an organic matter containing various functional groups such as amino, carboxyl and hydroxyl groups and can react with metal ions.^23–25^ It is the main cause of UF membrane fouling.^26–28^ In these circumstances, if PEUF is applied to drinking water treatment that deals with sudden heavy metal Cd pollution, it is necessary to investigate the capacity of the polymer to bind heavy metal ions and the influence of the feed water conditions on the process.

This research aimed to select a proper polymer to treat simulated Cd pollution in surface water during the PEUF process. Due to the importance of drinking water treatment technology to human health, the effects of the polymer, raw water and polymer recovery on the concentration of Cd in the permeate should be studied in a rigorous experiment. First, the performance of three polymers in dealing with Cd pollution was investigated, and a polymer with an excellent performance in treating simulated cadmium-contaminated raw water was subsequently selected. Second, the influences of the P/M, the solution chemicals and the HA content on the Cd removal rate in the PEUF process were investigated. In addition, transmembrane pressure differential (TMP) growth was used to analyze UF membrane fouling. Finally, the following processes were conducted: metal-containing polymer dissociation, polymer recycling, analysis the of efficiency of the recycled polymer, and proposal of a plan to deal with emergency Cd contamination in drinking water by applying PEUF technology.

## 2 Experiment

### 2.1 Materials

PAAS with a molecular weight of 4 × 10^6^ Da was obtained from the Shanghai Macklin Biochemical Technology Co. Ltd. (China). PVA with a molecular weight of 8.6 × 10^4^ Da was obtained from the Wuhan Zhuochuang Biochemical Co. Ltd. (China). CdCl_2_·2.5H_2_O, which was used to make the solution of Cd^2+^, was purchased from Tianjin Bodi Chemical Co. Ltd. HA was obtained from the Tianjin Guangfu Fine Chemical Research Institution. CTS with a molecular weight of 6.2 × 10^5^ Da and the other chemicals used in the experiments were obtained from Sinopharm Chemical Reagent Corp. (SCRC), China, unless otherwise noted. The water used in the experiments was deionized water (DI). The pH value was adjusted using NaOH or HCl as needed. The ionic strength of the solution was adjusted by adding an appropriate amount of NaCl. A flat sheet UF membrane with MWCO of 5 kDa was purchased from Shanghai Mosu Science Equipment Co. Ltd. (China) and a hollow fiber UF membrane with MWCO of 6 kDa was purchased from Dalian Yidong Membrane Engineering Equipment Co. Ltd. (China).

### 2.2 Experimental devices

**Fig. 1:**
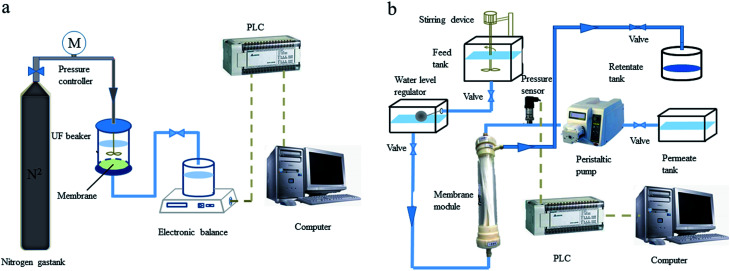
Schematic diagram of the ultrafiltration setups. (a) Flat sheet-type ultrafiltration membrane; (b) hollow fiber ultrafiltration membrane.

A laboratory scale UF system was employed in the experiments. There were two UF setups, the flat sheet-type UF membrane setup and the hollow fiber UF membrane setup. The available volume of the UF cup was small, and the cup was flexible for polymer selection. The hollow fiber UF membrane setup can more effectively and reliably simulate the UF treatment unit of a drinking water treatment plant. Thus, the hollow fiber UF membrane setup was applied in all PEUF experiments for removing Cd. The schematic diagram of the UF setups is shown in Fig. 1.

The flat sheet-type UF setup consisted of a UF cup, a pressure drive, an electronic balance and a counting system. The filtration area of the UF cup (Amicon-8400, Millipore, United States) was 41.8 cm^2^, and the volume was 400 mL. The filtration method of the flat UF membrane was dead end filtration. The UF experiment was driven by pure nitrogen gas with a constant pressure of 0.1 MPa. The hollow fiber membrane UF setup consisted of a hollow fiber membrane module, a high-water tank, a constant liquid level pool, a clear water pool, a concentrated liquid pool, a peristaltic pump, a pressure transmitter, a Programmable Logic Controller (PLC), a computer, *etc.* The Cd^2+^ and polymers reacted in the high-water tank. After being fully reacted, the solution flowed into the constant liquid level pool and entered the hollow fiber UF membrane module. The solution in the membrane module entered the clear water tank through the UF membrane driven by the peristaltic pump, and the concentrate liquid flowed into the concentrate liquid pool. The pressure transmitter was connected to the membrane module to record the TMP data of the hollow fiber membrane module in real time, and the data were uploaded to the acquisition system in the computer. The experiment was operated in constant flux mode, and the flux was controlled by the speed of the peristaltic pump. When the membrane module was being backwashed, only the peristaltic pump needed to be operated in reverse, the flux control was twice the forward running, and the flushing time was 1 min. The pretreatment methods for the two types of UF membrane were shown in ESI S1.†

### 2.3 Batch experiments

#### 2.3.1 Complexation reaction between Cd^2+^ and polymer

Before the complexation experiment, UF membrane with the MWCO of 50 kDa was used for purifying PAAS to remove the small molecular polymer reacting with Cd^2+^ until no PAAS was detected in permeable water (with TOC as index for PAAS). The Cd concentration in the experiment was based on the Cd pollution incident in the Longjiang River in China in 2012 and was close to 0.5 mg L^−1^. Therefore, the Cd concentration of the simulated Cd pollution was set at 0.5 mg L^−1^ in the experiments. The concentration mass ratio between the polymer and the Cd was expressed as the P/M in all experiments. In the polymer selection experiment, the solution pH was 7 when the effect of the P/M on the removal rate of Cd was studied, and when the effect of the solution chemicals on the Cd removal rate was studied, the P/M was 5, 10 and 30. In other experiments, mixed solutions of polymer and metal ions with different conditions (changing pH, Na^+^ concentration and HA, *etc.*) were prepared, and the P/M was 5, 10 and 30. Before the ultrafiltration experiment, the mixed solution of Cd^2+^ and one polymer under certain solution chemical conditions was gently agitated in the stirrer at a speed of 200 rpm for 1 h to ensure that the complexation reaction had reached equilibrium. After 1 hour, the UF experiment was conducted. To determine if the permeate could be used as drinking water, China's safe drinking water hygiene standards were used. These standards state that the Cd concentration in drinking water should be less than 0.005 mg L^−1^.

#### 2.3.2 UF experiment

The flat sheet-type UF membrane setup used was to select complexing agents. First, 1 L of deionized water was filtered, and then 350 mL of the mixed solution was filtered. When the volume of the permeate reached 300 mL, the experiment was stopped. The Cd^2+^ concentration in the permeate was determined. A new UF membrane was used for each set of experiments.

The hollow fiber UF membrane setup was used for treating surface water contaminated by Cd^2+^. Before each experiment, 1 L of deionized water was filtered to measure the TMP_0_ of pure water. When the TMP difference of pure water was large, the membrane was washed to restore TMP_0_. When investigating the effects of Cd removal rate under different conditions, 4 L of complexed solution was poured in the high-water tank. The flux was set as 7 L m^−2^ h^−1^, and the Cd^2+^ concentration in the permeate was measured. When the TMP increased to 75 kPa, the peristaltic pump operated in reverse and backwashed physically, then the peristaltic pump operated forward to filter 1 L of deionized water, and the TMP difference after backwashing was measured. Each set of experiments was conducted for 3 cycles.

Removal rate (*R*) of Cd was calculated by eqn (1):1
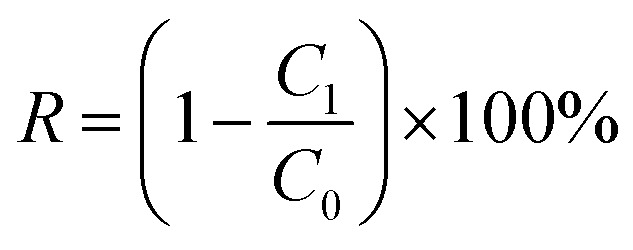
where *C*_1_ and *C*_0_ were the concentration (mg L^−1^) of Cd in the permeate and in feed water, respectively.

P/M was calculated as follows to indicate the concentration ratio of polymer to metal ion.2
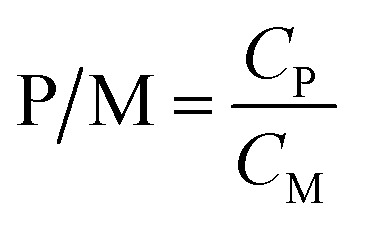
where *C*_p_ and *C*_M_ were the concentration (mg L^−1^) of polymer and the concentration (mg L^−1^) of Cd in feed water, respectively.

#### 2.3.3 Regeneration and reuse of polymers

In the neutral condition, the concentrations of Cd^2+^ and PAAS were 0.5 mg L^−1^ and 5 mg L^−1^, respectively, and the P/M was 10. The complexation process between Cd^2+^ and polymer was complete, and then the solution was circulated in the UF device until the Cd^2+^ concentration in the permeate was 0 mg L^−1^. Then, the quantitative (pre-prepared) HCl was quickly added to adjust the pH value to 2.5, and the dissociation experiment was started. Mechanical agitation was used to mix the solution quickly, and the concentration of Cd^2+^ in the permeate was measured continuously from 0 min (every 5 min). After the dissociation, the polymer was washed by deionized water with a pH of 2.5 until Cd^2+^ was undetected in the permeate. Finally, the PAAS solution was adjusted to neutral for the PAAS reuse experiment. The operating procedure of the reuse experiment was similar to the procedure outlined in Section 2.3.1.

### 2.4 Analysis

Generally, the constant flux filtration mode uses the curve of TMP growth with the filtration volume (or filtration time) to describe UF membrane fouling. The larger the TMP at the same filtration volume (or at the same filtration time), the greater the UF membrane fouling. The polymer concentration was determined with TOC as index for PAAS by using a total organic carbon analyzer (TOC-V_CPN_, Japan). The Cd concentration of the feed water and permeate was determined by using an ICP-AES (5300DV, USA). The solution pH value was determined by a pH meter (Lei-Ci, China). Scanning electron microscope (SEM) had been widely used for morphological analysis of membrane surface and cross sections. The samples were prepared in the following steps: (a) the fouled UF membrane samples were dried at room temperature for 48 h; (b) the UF membrane samples were cut into small pieces for the analysis of surfaces; (c) the UF membrane samples were frozen in liquid nitrogen and fractured for the analysis of cross sections; (d) then attached onto high purity aluminium specimen mounts by conductive resin; (e) then the UF membrane samples were sputter coated with a thin layer of gold in vacuum; (f) the surfaces and cross sections of UF membrane samples were imaged using SEM (Zeiss Sigma 500, GER) at appropriate magnification.

## 3 Results and discussion

### 3.1 Selection of complexing agent for PEUF technology

Drinking water treatment is related to the health and safety of people; in particular, the treatment of sudden heavy metal pollution should be more rigorous to examine the safety of each process. The first requirement for PEUF to remove heavy metals in drinking water is to select an excellent complexing agent. To determine the characteristics of polymers, which are safe, nontoxic and stable and can avoid disturbing the water quality conditions, three common polymers used in water treatment were selected for PEUF removal of Cd^2+^. The Cd removal efficiency of PEUF was examined based on the polymer dosage and solution chemistry, and finally, one polymer was selected as the complexing agent in PEUF.

**Fig. 2:**
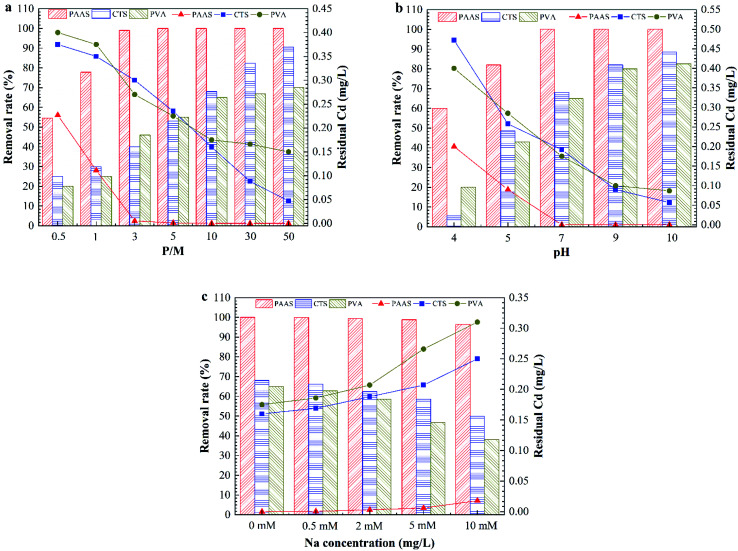
Effect of the P/M and solution chemistry on Cd removal rate by PEUF. (a) Effect of the P/M; (b) effect of the pH; (c) effect of the ionic strength.

Fig. 2 shows the effect of the P/M and solution chemistry on the removal rate of Cd^2+^ by PEUF. It was found that an increase in the P/M resulted in higher Cd^2+^ removal for all polymers. Obviously, PAAS performed better for removing Cd^2+^ from water by PEUF than PVA and CTS. For PAAS, the removal rate of Cd^2+^ reached 99% with a P/M value <5, but the Cd concentration in the permeate still exceeded the drinking water standard limit in China (0.005 mg L^−1^). When P/M ≥ 5, the Cd^2+^ concentration in the permeate was 0 mg L^−1^, and PEUF achieved 100% retention of Cd^2+^. For CTS, when the P/M increased to 50, the removal rate was 90.6%, and the Cd^2+^ concentration in the permeate was 0.047 mg L^−1^, which was still higher than 0.005 mg L^−1^. PVA had an inflection point when the P/M was 10. As the P/M continuously increased, the removal curve of Cd^2+^ was close to horizontal. The concentration of Cd^2+^ in the permeate was 0.175 mg L^−1^ under the maximum removal rate, which was significantly higher than the drinking water standard limit in China. It is notable that the removal of Cd^2+^ by PAAS and PVA reached the maximum when the P/M was 5 and 10, respectively. The removal rate of Cd^2+^ did not increase obviously when the P/M ≥ 10 for CTS-PEUF. Therefore, the effect of the chemical conditions on the removal of Cd^2+^ was studied when the P/M was 10. From Fig. 2b, it can be seen that the pH had a large effect on the removal rate of Cd^2+^. With the increase of the pH, the removal rate of Cd^2+^ increased for three polymers. Under the same pH, PAAS-PEUF had a higher removal rate of Cd^2+^ compared with the other two polymers and obviously resisted the pH fluctuation. When the pH of surface water fluctuated within 6–8, the removal rate of Cd^2+^ by the PAAS-PEUF system was higher than that of the other two processes. From Fig. 2c, when the ionic strength of solution increased, the removal rate of Cd^2+^ decreased for the three polymers, and the decline range of the PAAS-PEUF process was very small.

In the research above, the effects of different P/M values and solution chemicals on the removal rate of Cd^2+^ were analyzed. As a complexing agent to remove sudden heavy metal Cd pollution in surface water, PAAS has many advantages. The PAAS-PEUF system had a higher removal rate of Cd^2+^ compared with the other two polymers, and solution chemistry had relatively little effect on the removal rate of Cd^2+^ by PAAS-PEUF. PAAS was selected as the complexing agent. In the following part, the efficiency of PAAS-PEUF to remove Cd^2+^, the different influencing factors on UF membrane fouling, and the reuse efficiency of PAAS were studied in detail.

### 3.2 Study on the removal of Cd^2+^ by PAAS-PEUF

#### 3.2.1 Effect of the solution chemistry

In Section 3.1, the effects of the P/M and the chemical conditions on Cd^2+^ removal by PEUF were analyzed. PAAS was selected as the complexing agent. The effects of the solution conditions (pH, ionic strength) on Cd removal were studied for P/M values of 5, 10 and 30.

**Fig. 3:**
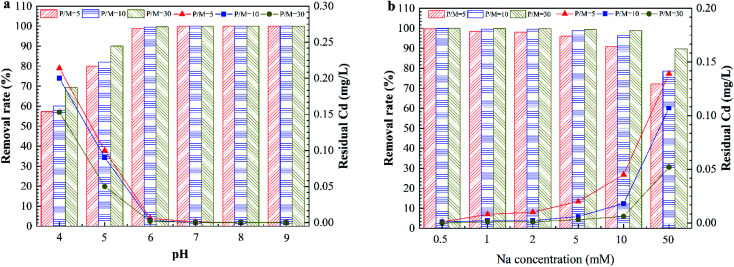
The effect of the solution chemistry on Cd removal by PAAS-PEUF. (a) The effect of pH; (b) the effect of ionic strength.

Fig. 3a shows the effect of the pH on Cd removal by PEUF when the P/M was 5, 10 and 30. The changing trend of the removal rate under three different conditions was the same as that when the pH value was increased. When the pH was greater than 8, the removal rate reached 100% for different P/M conditions. Fig. 3b shows the effect of the ionic strength on the removal rate of Cd^2+^. The removal rate decreased as the ionic strength increased. The concentration of Cd^2+^ was less than 0.005 mg L^−1^ in the permeate when the Na^+^ concentration was less than 0.5 mM for three different P/M conditions. When concentration of Na^+^ reached 1 mM and 2 mM, the Cd concentration in the permeate met the drinking-water standard in China with P/M values 10 and 30. However, the Cd concentration did not meet the standard in China (<0.005 mg L^−1^) with a P/M value of 5. When the concentration of Na^+^ was 5 mM, the Cd concentration in the permeate was 0.003 mg L^−1^ with a P/M value of 30. When the concentration of Na^+^ was 50 mM, the residual Cd concentrations for the three different P/M conditions exceeded 0.005 mg L^−1^. Generally, the Na^+^ concentration in surface water body is less than 1 mM, so the method can be applied in Cd removal in surface water with a P/M value of 10.

In general, the rate of a complexation reaction depends on the concentrations of the metal ions, ligand and hydrogen ions. When the solution was acidic, there was a large amount of H^+^ ions in the solution. H^+^ had a smaller radius, and the electron cloud had a higher density. The H^+^ ions more easily combined with –COO^−^, –O^−^ and –NH^−^.^29,30^ Therefore, the protonation reaction occurred in the complexation position and occupied the complexation position with Cd^2+^, which directly led to a significant reduction in the rejection of Cd^2+^. In contrast, in an alkaline environment, the deprotonation reaction with the complexing functional group caused more –COO^−^ functional groups to be exposed. In addition, when the pH was 10, Cd^2+^ also reacted with a large amount of OH^−^ and formed a hydroxyl compound. That was why the removal of Cd^2+^ significantly increased in the alkaline environment.^31,32^ As the ionic strength increased, a large amount of Na^+^ compressed the electric double layer of the organic molecules, resulting in molecular shrinkage.^33^ A large amount of Na^+^ occupied complexation sites that reacted with Cd^2+^ in organic molecules, which inhibited the interaction between Cd^2+^ and the complexation position.^21,34^ This interaction involved complexation and electrostatic adsorption between the complexing agent and Cd^2+^, which in turn reduced the removal of Cd^2+^ by the PEUF. The above experimental phenomena and analysis are helpful to better study the influencing factors to be considered in the process of Cd removal by PEUF and the corresponding measures.

#### 3.2.2 Effect of HA in feed water

There was a large amount of natural organic matter (NOM) in surface water. In the NOM, HA was a dominant component and contained a variety of functional groups with the ability to reach with metal ions. The HA was prone to compete with PAAS for Cd^2+^ complexation. The small HA molecules that reacted with Cd^2+^ would pass through the UF membrane,^35^ affecting the removal of Cd^2+^ by the PEUF process. At the same time, the existence of HA would aggravate the UF membrane fouling, affecting the efficiency of the membrane module and the clean frequency of the membrane.

**Fig. 4:**
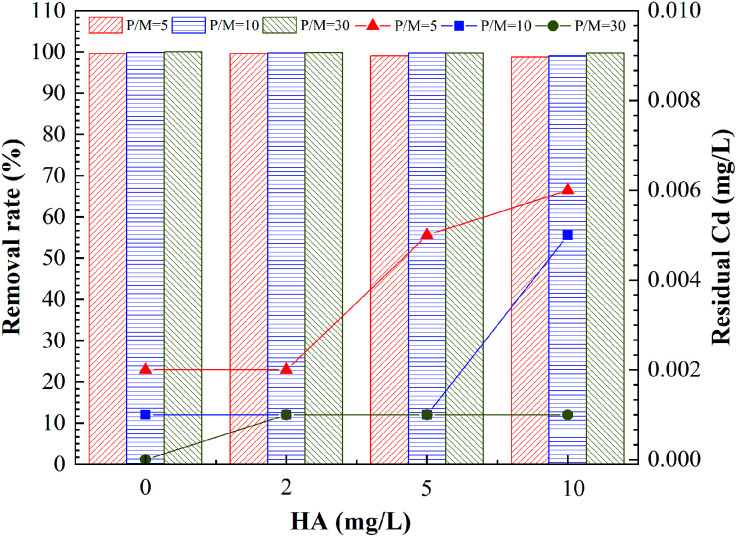
Effect of HA in the feed water on removal of Cd^2+^.

This study analyzed the effect of HA in the feed water on removal of Cd^2+^ by PEUF, as shown in Fig. 4.

The Fig. 4 shown the effect of HA on the removal rate of Cd^2+^ by PEUF. It can be observed from the figure that the removal rate of Cd^2+^ decreased as the HA increased. The larger the P/M value, the smaller the decrease in the Cd removal rate caused by the HA increase. When the HA in the feed water was 2 mg L^−1^, the Cd removal rate was greater than 99%, and the Cd concentration was lower than 0.005 mg L^−1^ in the permeate with P/M values of 5, 10 and 30, which could meet the safe drinking water standard in China. When the concentration of HA in the feed water was 5 mg L^−1^, the Cd concentration in the permeate with a P/M value of 5 was 0.005 mg L^−1^, which exceeded the safe drinking water standard. At the same time, the Cd concentration was 0.001 mg L^−1^ in the permeate with P/M values of 10 and 30. When the concentration of HA in the aqueous solution increased to 10 mg L^−1^, the Cd concentration in the permeate was lower than 0.005 mg L^−1^ with P/M value of 30.

Generally, HA molecules contain different functional groups that can react with metal ions, such as carboxyl groups, hydroxyl groups, and amino groups.^36^ These functional groups could complex with metal ions or interact with static electricity, so that metal ions bind to HA.^37–40^ The higher the concentration of HA, the stronger the interaction will be. Therefore, the existence of HA produced competition with PAAS, reducing the complexation rate between PAAS and Cd^2+^. The molecular mass distribution of HA was a discontinuous molecular interval, and small molecules of HA occupied for a large proportion. If the small HA molecules combined with Cd^2+^, then part of the HA–Cd that was smaller than the membrane pores and could pass through the UF membrane, thus affecting the removal of Cd^2+^ by PEUF process.

### 3.3 Analysis of UF membrane fouling

The ultrafiltration process in operation produced membrane fouling. Membrane fouling can be significant in PEUF to remove Cd pollution in surface water. Membrane fouling affected the flow rate of the permeate, thus, the removal efficiency of Cd^2+^.

#### 3.3.1 Effect of Solution Conditions on TMP

The P/M and solution pH were important factors that influenced the removal of Cd^2+^ and UF membrane fouling in the PEUF process. The UF membrane fouling is discussed in the following section.

**Fig. 5:**
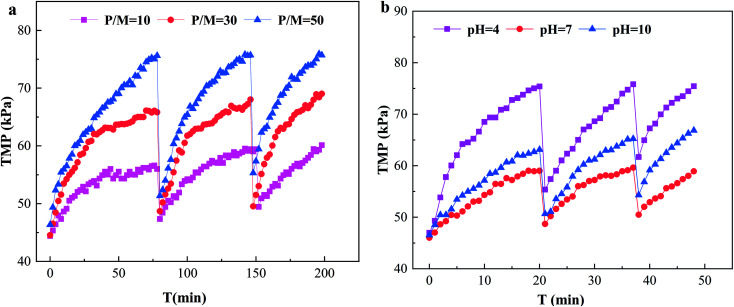
Effect of Solution Conditions on TMP. (a) The effect of P/M; (b) the effect of pH.

Fig. 5a shows the effect of P/M on TMP in the PEUF process. Obviously, the larger the P/M value was, the faster the TMP increased; in particular, the TMP with a P/M of 50 in the first cycle reached 75.62 kPa at filtration time of 78 min (as explained in the experiment section, filtration was stopped when the TMP increased to 75 kPa), and the TMP with a P/M of 10 and 30 was 56 kPa and 65 kPa, respectively, at the same time. After hydraulic backwashing, the initial TMP in the second cycle increased slightly compared with that in the first cycle, indicating that irreversible fouling was generated after 1 cycle. The time taken for TMP to reach 75 kPa in cycles 2 and 3 with a P/M of 50 was shortened. For the processes with P/M values of 10 and 30 at the same operating time, the TMP increase was less than that with a P/M of 50. Obviously, in the system with a large P/M, the PAAS concentration was higher, and the accumulated fouling layer on the membrane surface was thicker under the condition of the same permeation flux, and the increase rate of TMP was accelerated, which was the reason for the rapid rise of TMP with the increase of the P/M. In addition, PAAS also interacted with the surface of the UF membrane and was adsorbed by the surface of the UF membrane. This was the reason why the TMP was higher than that of the new membrane after hydraulic backwashing and why the initial TMP increased gradually with the increase of the filtration period.

The effect of the solution pH on TMP is shown in Fig. 5b. From the figure, the TMP with a pH of 4 increased more quickly, and the TMP reached the limit set by the experiment at 20 min after UF, while the TMP with a pH of 10 was larger than with a pH of 7. The times for the pH 4 process to reach 75 kPa in cycles 2 and 3 were shortened to 17 min and 11 min, respectively. From the figure, the UF membrane fouling was quite serious under acidic conditions, and the UF membrane fouling was greater at a pH of 10 than at a pH of 7. This behavior results from the fact that the change of the acidity and alkalinity of the solution seriously affected the molecular configuration and existing state of PAAS and Cd^2+^, thus affecting the UF membrane fouling. When the pH was acidic, the –COO– in the PAAS molecule was protonated, the net charge of the molecule was reduced, and the surface electronegativity was reduced,^33^ resulting in an increase in the aggregation of PAAS molecules, which deposited on the surface of the UF membrane, formed a filter cake layer and aggravated the UF membrane fouling. On the other hand, the UF membrane was negatively charged, so the interaction between PAAS and the UF membrane was reduced, and its adsorption on the UF membrane surface was enhanced,^41^ which aggravated the UF membrane fouling. When the pH was 10, the Cd^2+^ in the solution reacted with OH^−^ to form slightly soluble Cd(OH)_2_, and the combination of Cd(OH)_2_ and PAAS aggravated the UF membrane fouling.

#### 3.3.2 Effect of HA on TMP

**Fig. 6:**
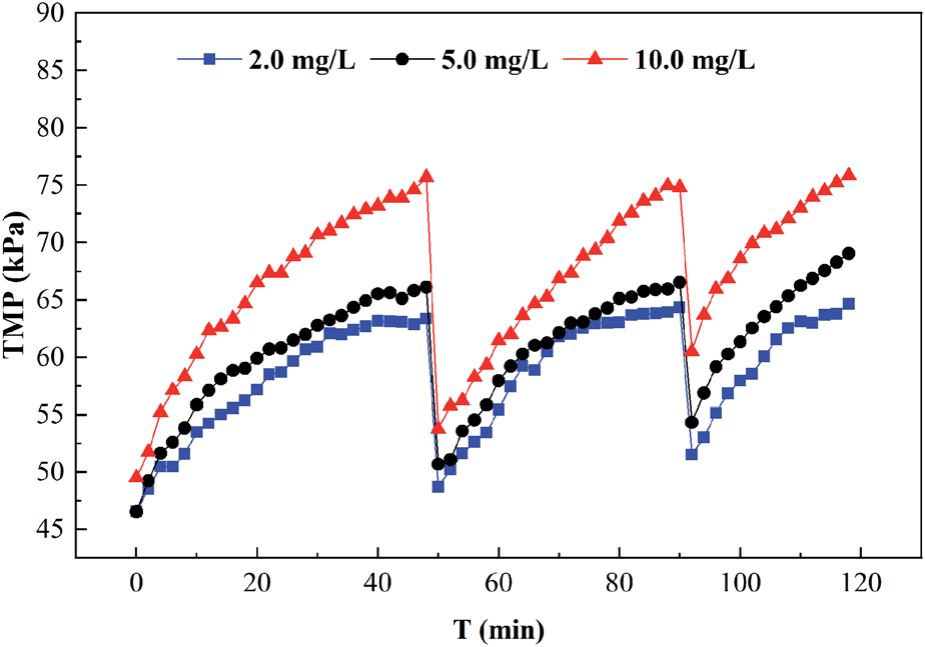
Effect of HA on TMP.

Fig. 6 shows the effect of HA on TMP. It can be seen from the figure that the increase rate of TMP was accelerated with the presence of HA in the aqueous solution; in particular, the higher the concentration of HA was, the faster the increase rate of TMP was. It could also be observed that with the increase of filtration cycle hydraulic backwashing after the initial TMP, the TMP was larger than that of pure water. It was obvious that for an HA concentration of 10 mg L^−1^, thirdly cycle initial TMP reached 60 kPa. Severe membrane fouling was caused by the high concentration of HA in feed water, which was generally difficult to remove by hydraulic backwashing. Hence, the increase of hydraulic irreversible fouling was larger, and chemical cleaning was needed at this time.

HA is the main substance that causes membrane fouling.^42,43^ UF membrane fouling by HA is mainly caused by pore constriction, membrane pore blocking and cake layer fouling. On the one hand, HA with a small molecular size easily entered the inside of the membrane pores and adsorbed on the pore walls, resulting in narrow pores. The molecular weight of the HA was equal to that of the membrane pores so the membrane pores were plugged by the HA,^26^ which was generally difficult to remove by hydraulic cleaning. On the other hand, the HA with a macromolecule weight mainly caused cake layer fouling due to simultaneous adsorption and membrane pore blockage. When the cake layer was formed, the UF membrane fouling was mainly cake layer fouling, which could generally be removed by hydraulic cleaning. The UF membrane fouling by PAAS molecules was mainly cake layer fouling, and this irreversible fouling increased greatly in the presence of HA, as shown in Fig. 6. Therefore, for the removal of Cd^2+^ by the PEUF process, the presence of HA not only affected the removal rate of Cd^2+^ but also seriously affected the UF membrane fouling. The application of the PEUF process to the actual process requires a focus on the influence of the presence of HA. The solution is to consider installing a pretreatment process at the front end of the PEUF process to remove HA and other interfering substances.

#### 3.3.3 Analyses of SEM images of membrane fouling

**Fig. 7:**
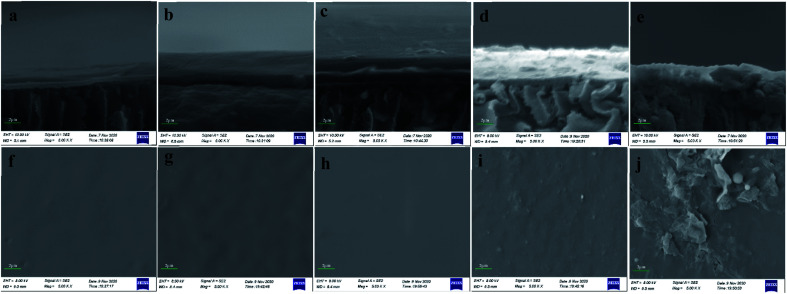
SEM images of cross sections of membrane: (a) P/M = 10, (b) pH = 4, (c) P/M = 50, (d) HA = 2 mg L^−1^, (e) HA = 10 mg L^−1^ and SEM images of surfaces of membrane: (f) P/M = 10, (g) pH = 4, (h) P/M = 50, (i) HA = 2 mg L^−1^, (j) HA = 10 mg L^−1^.

Fig. 7 shows the SEM images of surfaces and cross sections of fouled membrane at P/M = 10, pH = 4, P/M = 50, HA = 2 and HA = 10.

It is obvious that there was a thick layer of foulants on the surface of UF membrane (Fig. 7a). And the surface of fouled membrane was smooth without any particulate material and pore in SEM image under 5000 magnification times (Fig. 7f). Fig. 7b shows the SEM image of cross sections of fouled membrane at pH 4. We can see that the layer of foulants was thick and combined with membrane closely at acidic pHs, and the UF membrane samples were not fractured regularly after being frozen in liquid nitrogen for 20 min. From Fig. 7g, the surface of fouled membrane at acidic pHs was more rough compared with that at neutral pH. This is because the protonation reaction of carboxyl groups in PAAS at acidic pHs decreased electric charge, which reduced the repulsive force between PAAS and resulted in the aggregation of PAAS. Moreover, the hydrophobicity of PAAS increased at acidic pHs, and thus the interaction between PAAS and UF membrane was enhanced, which accelerated the deposition process of PAAS on the surface of UF membrane. Fig. 5b also accounted for the serious membrane fouling at pH 4. Fig. 7c and h exhibits SEM images of cross sections and surface of UF fouled membrane at P/M = 50, respectively, according to which the layer of foulants was thick and the surface of UF membrane was smooth. This is due to the high concentration of PAAS in raw water at P/M = 50. Fig. 7d and e shows SEM images of cross sections of UF fouled membrane at HA = 2 mg L^−1^ and HA = 10 mg L^−1^, respectively. It can be seen that the condition of UF membrane fouling varied in the presence of HA. And there were particulate materials on the surface of UF membrane and foulants in the pore of UF membrane according to Fig. 7i and j. Especially at HA = 10 mg L^−1^, the pore of UF membrane was blocked, and the particulate materials on the surface of UF membrane also increased. The reason for this phenomenon is that HA is a substance with a wide molecular weight distribution, among which the small molecular can further decrease the porosity of UF membrane after being adsorbed, the molecular with similar size to the pore of UF membrane will block these pores directly, and the large molecular is retained on the surface. Meanwhile, it is difficult to remove the foulants in the pore of UF membrane completely through conventional hydraulic washing, and thus chemical cleaning is needed. Therefore, the presence of HA will affect the cadmium removal by PEUF adversely.

### 3.4 Polymer regeneration and utilization efficiency

**Fig. 8:**
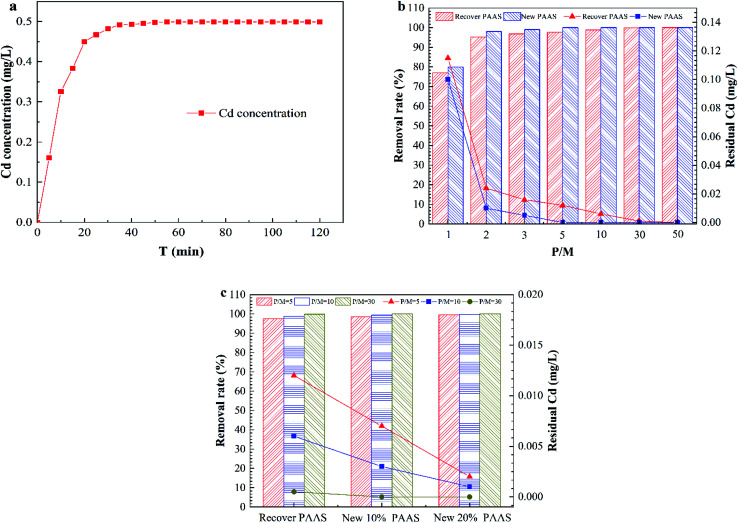
The dissociation efficiency of PAAS–Cd and the performance of the regenerated PAAS. (a) the concentration of Cd in the permeate; (b) the removal rate of new and regenerate PAAS; (c) the removal rate of Cd^2+^ after 10% and 20% of new PAAS was added to the regenerated PAAS.

Fig. 8 shows the dissociation efficiency of PAAS–Cd and the performance analysis of the dissociated regenerate PAAS for PEUF.

The complexing agent PAAS was purify by UF, and the permeate did not contain PAAS, so it was difficult to determine the degree of dissociation according to the concentration of PAAS. Therefore, the dissociation efficiency of PAAS–Cd under acidic conditions could only be determined by detecting the concentration of Cd^2+^ in the permeate. The Fig. 8a shows the variation of Cd^2+^ concentration in the permeate with time for a solution pH of 2.5. From Fig. 8a, at a pH of 2.5, the PAAS–Cd complex dissociation reaction was a quick process, and within 20 min the concentration of Cd^2+^ in the permeate increased linearly. When the dissociation time was 50 min, the Cd^2+^ concentration was 0.499 mg L^−1^, and then within 120 min the Cd^2+^ concentration in the permeate was constant, and the dissociation reaction was complete. Fig. 8b compares the removal of Cd^2+^ with the use of recyclable PAAS and new PAAS by PEUF. From the figure, the removal rate of Cd^2+^ increased as the P/M increased, but the recovered PAAS could not make the permeate meet the drinking water standard in China when the PM ≤ 10 (the Cd concentration in the permeate was 0.006 mg L^−1^), while the new PAAS could meet the drinking water standard when the P/M was 5 (the Cd concentration was 0 mg L^−1^). Only when the P/M of recycled PAAS was 30 was the Cd concentration lower than 0.001 mg L^−1^ in the permeate. Fig. 8c shows the change diagram of the removal rate of Cd^2+^ after 10% and 20% of new PAAS was added to the recycled PAAS. From the figure, after 10% of new polymer was added to the recycled PAAS, the permeate with P/M values of 10 and 30 met the drinking water standard, while the permeate with P/M values of 5, 10 and 30 met the drinking water standard after 20% of new PAAS was added. Thus, the recovered PAAS can be used in the PEUF process to remove Cd after a certain amount of new PAAS is added.

### 3.5 Feasibility evaluation of Cd pollution treatment by PEUF

The above experiments confirmed that the removal rate of Cd^2+^ by PEUF was higher than 99% under ideal conditions and the permeate could be used in drinking water. However, there were many interfering factors when PEUF treated actual heavy metal pollution in surface water; therefore, the influence of the solution chemistry and HA concentration on the removal rate of Cd^2+^ by PEUF was investigated in the experiment. The experimental results show that pH was the main factor affecting the removal efficiency of Cd^2+^. Fortunately, the pH of surface water generally varies within 6–8, and there is no need to adjust the pH in actual drinking water treatment plants. The presence of HA in the experiment affected the removal rate of Cd^2+^ and aggravated the UF membrane fouling. Surface water contains organic materials such as HA, which was the key factor to be considered in the PEUF process. The feed water solution is to consider pretreatment of the raw water before the PEUF process to remove organic and inorganic substances. Most drinking water treatment plants still use coagulation and other process combinations, such as coagulation/activated carbon adsorption, coagulation/ozone/activated carbon, coagulation/ultrafiltration and ultrafiltration processes. On the one hand, these pretreatment processes remove organic substances such as HA and inorganic substances; on the other hand, they can reduce the concentration of heavy metals to a certain extent. The dissociation rate of complex PAAS–Cd was over 99.8%, and the PAAS repetition rate was good. The high removal rate of Cd^2+^ and the high dissociation rate of PAAS–Cd complex were the basis for further study and promotion by PEUF to remove heavy metal pollution. When heavy metal pollution in surface water occurred, the pollution speed was very fast, and it was difficult to determine the pollution through observation. Generally, the pollution was detected after a period of time. Therefore, the drinking water treatment plant needs to make a rapid emergency treatment plan, which requires the removal of heavy metals as quickly and effectively as possible. Heavy metal removal processes need to be combined with existing processes in current drinking water treatment plants to minimize the need for new treatment structures. The UF process has the advantages of a good treatment effect for pollutants, a small floor space, a fast construction process, and good matching with coagulation processes. Therefore, it is feasible to apply enhanced ultrafiltration technology to remove heavy metal pollution in drinking water.

The treatment of heavy metal pollution by PEUF technology is a new research idea. This experiment research heavy metal pollution in water sources has great significance; at the same time, the experimental results also reveal the main limiting factor of PEUF in removing heavy metal pollution, and this need continued in-depth studies combined with experiments with actual water bodies. This study provides a reference for the treatment of heavy metal pollution in water sources.

## 4 Conclusions

The treatment of sudden Cd pollution in surface water by PEUF was investigated. A comprehensive analysis of the influence of the P/M, pH and ionic strength on the removal of Cd^2+^ by PEUF using CTS, PVA and PAAS as complexing agents was conducted. PAAS was selected as the complexing agent of the three polymers for PEUF based on the removal rate of Cd^2+^. At optimum experimental conditions, the removal rate of Cd^2+^ reached 100.0% using PAAS as the complexing agent. The solution pH was found to be the major factor that determines the removal rate of Cd^2+^ using the PEUF process. When the pH was more than 8, the removal rate reached 100% for different P/M values. When the ionic strength and HA concentration were lower, the removal rate of Cd^2+^ was higher. The analysis showed that the mechanism of ionic strength could be a compressed electric double layer, and the mechanism of HA and H^+^ was competitive complexation. When the P/M was larger, the pH was lower and the HA concentration was higher, the UF membrane fouling was more severe. At a pH of 2.5, the dissociation rate of PAAS–Cd reached 99.8%. When P/M ≤ 10 and PAAS was regenerated, the Cd concentration in the permeate was larger than 0.005 mg L^−1^; when the P/M was 5, 10 and 30 and 20% new PAAS was added in the regenerate, the Cd concentration was lower than 0.005 mg L^−1^ in the permeate. The PEUF process is viable and suitable to remove Cd pollution in surface water, and the research provides a theoretical and technological reference for dealing with sudden Cd pollution in surface water.

## Conflicts of interest

No conflict of interest exits in the submission of this manuscript, and manuscript is approved by all authors for publication.

## Supplementary Material

RA-011-D0RA10818A-s001
