# Supplementary material for: Study on the treatment of sudden cadmium pollution in surface water by a polymer enhanced ultrafiltration process
Source: RSC Adv. 2021 Feb 12;11(13):7405–15. doi: 10.1039/d0ra10818a (PMC8695008; doi:10.1039/d0ra10818a)
Supplement: RA-011-D0RA10818A-s001 [file RA-011-D0RA10818A-s001.pdf]

Electronic Supplementary Material (ESI) for RSC Advances.
This journal is © The Royal Society of Chemistry 2021

At first two kinds of UF membranes were needed to be pretreated to remove protective components. The flat sheet-type UF membrane was firstly immersed in alcohol for 15 minutes, then washed by deionized water and soaked for 1 hour. The operation was repeated 3 times until no TOC was detected. In the end the membrane was soaked in deionized water and placed in the refrigerator for storage. Hollow fiber UF membrane was pretreated by direct flushing, cyclic cleaning, and immersion cleaning. Firstly, the inlet was filled with deionized water and operating in the low pressure (<0.1MPa). The concentrate solution and permeate were directly discharged outward, and the deionized water was continuously injected and directly discharged for 5 minutes. Then, the concentrated solution was refluxed into the original liquid tank and operating in the low-pressure circulation for 2 minutes. The concentrated liquid was directly discharged to the outside. Finally, the concentrated liquid was returned back to the tank, and the lower pressure operation lasted for 2 minutes. After shutdown, the membrane was immersed in DI water for 24 hours.
